# Research Progress on the Detection Methods of Botulinum Neurotoxin

**DOI:** 10.3390/toxins17090453

**Published:** 2025-09-08

**Authors:** Shuo Wang, Huajie Zhang, Yanhua Xue, Yingchao Yang, Liyong Yuan

**Affiliations:** National Institutes for Food and Drug Control, Beijing 102629, China; wangshuo@nifdc.org.cn (S.W.); zhanghj@nifdc.org.cn (H.Z.); xueyh1999@163.com (Y.X.); yangyc@nifdc.org.cn (Y.Y.)

**Keywords:** botulinum neurotoxins, detection methods, cell-based assays, endopeptidase–mass spectrometry, biosensors

## Abstract

Botulinum neurotoxins (BoNTs), produced by the anaerobic spore-forming bacterium Clostridium botulinum, are among the most potent known biological toxins. BoNTs cause lethal botulism via contaminated food, wound infections, or infant intestinal colonization, posing significant threats to public health. Although the mouse bioassay is still being considered as the gold standard for detecting BoNTs, its drawbacks, including the lengthy experimental duration, high costs, and ethical issues, highlight the urgent need to develop alternative methods to fulfill the detection requirements. In recent years, frequent botulism poisoning incidents haves put forward higher requirements for detection technology. On-site detection is expected to be rapid and immediate, while laboratory detection requires high sensitivity and serotype discrimination capabilities. This review comprehensively introduces current detection approaches, including mouse bioassay, cell-based assays, immunological methods, endopeptidase–mass spectrometry, biosensors, chromatography, and mass spectrometry techniques. Notably, cell-based assays have been used for the potency testing of commercialized botulinum toxin type A and are considered the most promising alternative to the mouse bioassay. Biosensors based on nanomaterials demonstrate advantages in real-time detection due to their rapid response and portability, while endopeptidase–mass spectrometry achieves high sensitivity and effective serotype identification by specifically recognizing toxin-cleaved substrates. Future works shall aim to completely replace MBA, developing a detection system suitable for multiple scenarios such as clinical diagnosis, food safety monitoring, and environmental monitoring. The detection methods should also have matrix compatibility and serotype discrimination capabilities.

## 1. Introduction

**Figure 1:**
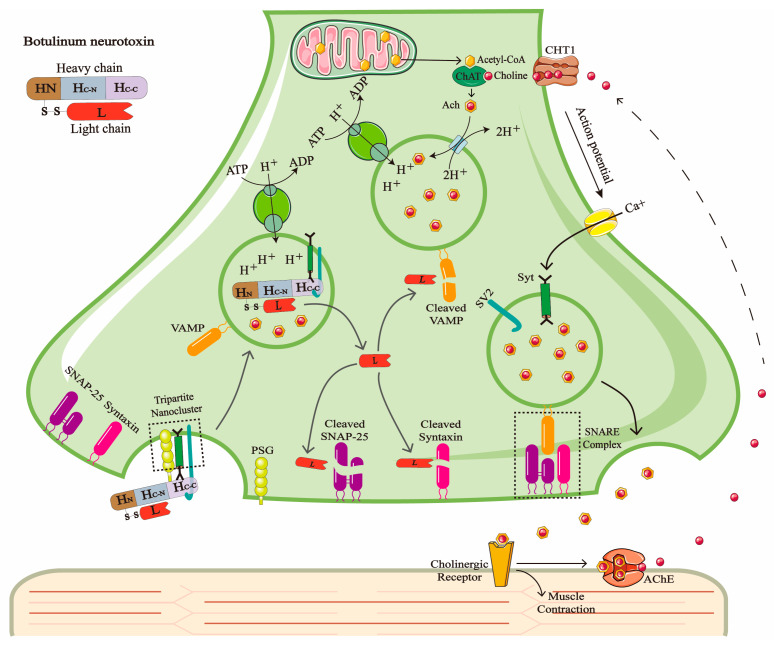
The synthesis and release of ACh and the toxic mechanism of BoNT. ACh is a neurotransmitter that mediates signaling between cholinergic motor neurons and muscles. Its synthesis is catalyzed by choline acetyltransferase (ChAT) in the cytoplasm, which condenses choline with acetyl-CoA. Subsequently, the V-ATPase on the synaptic vesicle membrane uses the energy from ATP hydrolysis to pump H^+^ into the vesicle, creating an acidic environment that drives ACh to enter the vesicle against its concentration gradient. When an action potential reaches the presynaptic terminal, the influx of Ca^2+^ binds to synaptotagmin, triggering the fusion of synaptic vesicles with the plasma membrane mediated by SNARE complexes, thereby releasing ACh into the synaptic cleft through exocytosis. After ACh binds to the postsynaptic membrane receptors and induces muscle contraction, it is rapidly degraded by acetylcholinesterase (AChE). The released choline is then reuptaken into the presynaptic membrane via the choline transporter (CHT1) and combined with mitochondrial-derived acetyl-CoA to participate in the next round of ACh synthesis. BoNT binds to the PSG on the presynaptic membrane through its heavy chain C-terminal domain (HC-C) and subsequently binds to Syt and SV2. The toxin is internalized via a tripartite nanocluster-mediated mechanism. In the acidic vesicular environment, the LC translocates into the cytoplasm and specifically cleaves SNARE proteins (such as SNAP-25, syntaxin, or VAMP) depending on the toxin serotype. This blocks the vesicle fusion process and ultimately inhibits the release of ACh, leading to muscle paralysis.

BoNT is a type of zinc finger metalloproteinase produced by a Gram-positive, anaerobic, spore-forming Clostridium microbe with extremely high neurotoxicity. BoNTs consist of two chains, a 100-kDa heavy chain (HC) responsible for transport and a 50-kDa light chain (LC) that exerts enzymatic activity, and the two chains are connected by disulfide bonds [1,2,3]. The HC binds to a tripartite nanocluster on the neuronal plasma membrane, which is composed of polysialoganglioside (PSG), synaptotagmin (Syt), and synaptic vesicle glycoprotein 2 (SV2), mediating the transport of LC into the cell [4,5,6]. The LC can specifically cleave the soluble N-ethyl maleimide-sensitive factor attachment protein receptor (SNARE) within the neuron. SNARE is a type of protein that plays a key role in the process of intracellular membrane fusion, including vesicle-associated membrane protein (VAMP), syntaxin, and synaptic vesicle-associated protein 25 (SNAP-25), thereby blocking the release of the neurotransmitter acetylcholine (Ach) (Figure 1) [7,8]. The clinical symptoms of botulism include muscle paralysis, blurred vision, and difficulty with speech and swallowing. In severe cases, it can lead to respiratory failure and death. BoNTs are classified into seven serotypes, A–G, according to the specific types of antisera by which they are neutralized. Different serotypes of BoNTs can cleave different proteins in the SNARE family at different sites. SNAP-25 is mainly cleaved by BoNT/A, C, and E at sites 197–198, 198–199, and 180–181, and syntaxin is cleaved by BoNT/C at site 253–254. VAMP is cleaved between amino acid pairs at positions 76–77, 59–60, 58–59, and 81–82 by serotypes BoNT/B, D, F, and G. BoNT/A, B, E, and F mainly cause botulism in humans, while BoNT/C and D are mainly related to botulism in animals [1,9].

Although botulism outbreaks are relatively rare under modern food hygiene systems, the neurotoxin produced by the ubiquitous soil bacterium Clostridium botulinum remains a persistent public health and food safety hazard. Its threat lies not in widespread transmission but in its extremely high toxicity and diverse exposure routes. Foodborne botulism remains the most common route of human infection, and the chief culprit is usually home-canned or home-fermented foods. Whether it is home-cured ham, handmade sausage, smoked fish, or fermented seafood, once a sealed, oxygen-poor environment is created, spores of Clostridium botulinum readily germinate and produce a toxin, setting the stage for an outbreak [10,11,12]. Infant botulism poisoning is different from foodborne botulism poisoning. Infants with immature intestinal microbiota ingest botulism spores rather than toxins. Once these spores enter the baby’s intestines, they will germinate, colonize, grow, and start to produce toxins. Honey is now regarded as the most common food carrying these spores [13,14,15]. Another approach is through wound infection. When the wound is contaminated by soil or dust containing Clostridium botulinum spores and an anaerobic environment is formed inside the wound, bacteria can produce toxins within the wound, which is similar to the mechanism of tetanus infection [16]. The primary medium for animal botulism transmission is water. When decaying organic matter from plants and animals enters lakes or ponds, it can trigger massive bacterial proliferation and toxin production in the water. Birds then ingest these toxins while feeding, leading to mass mortality events [16]. There are also cases where artificially farmed animals get poisoned by consuming feed contaminated with toxins, which occurs from time to time in the livestock industry [17]. BoNTs are mainly used in clinical treatments of diseases such as dystonia, strabismus, and multiple sclerosis. The inhibitory effect of BoNTs on Ach release in neurons can reduce muscle activity and reduce facial wrinkles, which has led to its increasing use in the field of medical aesthetics [18,19,20]. However, excessive injection of BoNTs can cause iatrogenic botulism [21,22]. Currently, botulism treatment relies on antitoxin administration and supportive care, as no effective antidotes exist, while established neural damage is irreversible. According to epidemiological data from multiple countries, although botulism has a relatively low overall incidence rate, the fatality rate after poisoning is high, and it is often misdiagnosed. For instance, the misdiagnosis rate of foodborne botulism poisoning incidents in China from 2004 to 2020 reached 27.5% [23]. From 1997 to 2019, Spain also reported multiple cases that were misdiagnosed as other diseases such as Guillain–Barre syndrome and myasthenia gravis; the laboratory diagnosis rate of 232 cases of botulism poisoning was only 33.2% [24]. Canadian data shows that 78% of patients need to be transferred to special care units, 70% require mechanical ventilation, and the hospital stay can last for several months [11]. In addition, the clinical manifestations and severity caused by toxins of different serotypes vary significantly, further increasing the complexity of diagnosis. Global data shows that among 2943 cases of infant botulism from 2007 to 2021, the serum types of toxins in 36 cases were not identified [14]. There has been a long-term underreporting of cases in underdeveloped regions such as Africa. Early use of antitoxins is the key to improving prognosis, but its effectiveness highly depends on timely and accurate diagnosis. Therefore, developing and promoting rapid, sensitive, and specific BoNT detection methods, especially in grassroots and remote areas, is of vital significance for improving diagnostic accuracy, reducing misdiagnosis, and ensuring that patients receive effective treatment in a timely manner.

The existing methods for detecting BoNTs include mouse bioassay (MBA), cell-based assay (CBA), immunological detection, endopeptidase–mass spectrometry (Endopep-MS), and biosensor. Different detection methods have their own advantages and limitations. MBA is regarded as the “gold standard” for detecting BoNTs, but its limitations in terms of ethics and cost have prompted researchers to constantly explore alternative methods [25,26]. While CBA effectively simulates BoNT intoxication in neuronal cells, its utility is limited by potential matrix interference and the requirement that samples must be kept sterile during culture [27,28,29,30]. Immunological detection methods, such as enzyme-linked immunosorbent assay (ELISA), have attracted much attention due to their simple operation and low cost, but their performance is highly dependent on the development and application of specific antibodies [31,32]. In addition, some emerging detection technologies, such as electrochemical sensors and nanotechnology-based biosensors, have been applied alone or in combination with other methods for the detection of BoNTs [33,34,35,36]. These novel methodologies aim to develop rapid, sensitive, and cost-effective BoNT detection platforms addressing escalating food safety and public health requirements.

In conclusion, the detection and research of BoNTs is an interdisciplinary field that involves microbiology, molecular biology, immunology, neuroscience, and public health. Future studies are expected to focus on improving the accuracy and accessibility of detection techniques while advancing the understanding of biological characteristics of BoNTs to promote the development and application of new treatment methods.

## 2. Mouse Bioassay

MBA is widely recognized as the “gold standard” method for detecting BoNTs due to its outstanding reliability, sensitivity, quantitative capabilities, and comprehensive coverage of all BoNT serotypes. Intraperitoneal injection of BoNT causes characteristic flaccid paralysis in mice due to the inhibition of Ach release at the neuromuscular junction (NMJ). The pathological changes manifested include abdominal weakness, a waist-like body shape, breathing difficulties, and eventually death due to respiratory failure. The quantitative endpoint of MBA is mortality, and efficacy is calculated with the mouse LD50 bioassay (mLD50) [25]. It can accurately identify BoNTs with biological activity and is suitable for detection in a variety of different matrices [25,37]. However, challenges persist in the application of MBA. First of all, lethality in animal experiments not only triggers ethical controversies but also increases experimental costs [38]. Secondly, MBA execution necessitates dedicated experimental facilities, trained personnel, prolonged processing time, and stringent safety measures against toxin exposure. Moreover, MBA results exhibit sensitivity to multiple variables including sample preparation, operator injection technique, as well as the age and strain of animals. Therefore, rigorous control of experimental conditions is essential to guarantee result accuracy and reproducibility.

To further enhance the accuracy of the experimental results, various measures are adopted. For instance, the use of standardized toxin references to determine BoNT activity and specific buffers (e.g., gelatin buffers) to stabilize BoNT have effectively reduced experimental variability and uncertainty [39]. Researchers also continue to explore alternatives to reduce, refine, or replace animal testing. Current partial solutions like the mouse phrenic nerve-hemidiaphragm detection and low-dose non-lethal injection methods aim to mitigate animal suffering and ethical concerns while still requiring live animals [40,41,42]. For non-lethal doses or studies requiring the assessment of local effects, a functional scoring system can be adopted, including quantifying local muscle paralysis of the hind limb using the Digit Abduction Score (DAS) and observing the severity and occurrence time of progressive paralysis symptoms for scoring [43]. Technological progress enables exploration of alternative BoNT detection technologies that comply with ethical guidelines while maintaining testing efficacy, aiming to reduce dependencies on conventional MBA methods.

## 3. Cell-Based Assay

At present, CBA has demonstrated a significant advantage in the field of BoNT detection. This method can rapidly and quantitatively detect the activity of toxins in vitro and can fully simulate the molecular toxicity mechanism of BoNTs in vivo, including the specific binding of HC to the surface receptors of nerve cells, the receptor-mediated endocytosis process, the entry of LC into the cytoplasm, and the LC-catalyzed SNARE protein hydrolysis [25]. The cleaved peptides generated by this proteolysis process are regarded as key molecular markers. BoNT/A, C, and E cleave SNAP-25; BoNT/B, D, F, and G cleave VAMP, while BoNT/C cleaves Syntaxin. To quantify BoNT-mediated SNARE cleavage, techniques such as Western blotting, ELISA, or MS are typically employed to detect the relative abundance of cleavage products and intact SNARE proteins, respectively. In addition to detecting the cleavage products, CBA can also be combined with electrophysiology to assess the toxicity of BoNT by detecting the spontaneous network bursts and the degree of inhibition of neurotransmitter release [44,45]. Compared to MBA, CBA enables stage-specific analysis of BoNT toxicological mechanisms, overcoming corresponding limitations of animal experiments. The total avoidance of live animal experiments allows CBA to adhere to the “3R principle” and avoid ethical controversies. High-throughput CBA detection can be made possible through large-scale preparation of cell models, strongly supporting rapid detection and diagnosis of BoNTs. Limitation of the CBA include incomplete simulation of BoNT pharmacokinetics in vivo [46] and the strict requirements for the sample matrix, as CBA is highly sensitive to the matrix effect when detecting environmental or food samples. In general, CBA may potentially replace MBA as the main method for the detection of BoNTs due to its rapid, efficient, sensitive, and ethically acceptable advantages. The feasibility of this method for detecting BoNTs has received extensive literature support in terms of mechanism research and practical applications [25,27,30,47,48].

When employing CBA for the detection of BoNTs, selection of appropriate cell types emerges as the primary determinant. In previous studies, three types of cell systems were mainly adopted—continuous cell lines, primary neurons, and embryonic stem cells—each demonstrating characteristic technical advantages and limitations. Continuous cell lines, such as PC12, SH-SY5Y, and SiMa cells, are widely used in related research due to their strong renewability, cost-effectiveness, and operation simplicity [49,50,51]. The PC12 cell line, derived from pheochromocytoma, exhibits low sensitivity to BoNTs, responding only at the nanomolar level, and typically requires a prolonged incubation period (48–72 h) to detect toxic effects [52]. SH-SY5Y cells show significant variation in sensitivity to different BoNT serotypes: they exhibit moderate sensitivity to BoNT/A and BoNT/B and the highest sensitivity to BoNT/C. It is notable that the sensitivity of primary rat spinal cord neurons to BoNT/A is approximately 200,000 times higher than that of SH-SY5Y cells. This difference is not determined by SNARE protein substrate specificity but is likely related to the mechanisms by which different serotypes enter cells. The highly sensitive BoNT/C may efficiently enter SH-SY5Y cells via receptor-mediated endocytosis, whereas other serotypes may display lower sensitivity due to the lack of a corresponding efficient cellular entry approach [51]. In contrast, SiMa cells demonstrate high sensitivity to BoNT/A and have been employed as a cellular model in detection of commercial BoNT/A products [53]. SiMa cells not only responds well to BoNT/A but also shows good detection capability for BoNT/B, with sensitivity comparable to the MBA [49]. Its detection sensitivity for BoNT/E even surpasses that of the MBA [29]. Primary neurons are usually a mixture of neurons and supporting cells extracted from the nervous system of newborn animals. Obtaining primary neurons requires animal sacrifice and technical expertise but enables region-specific enrichment of neurons in the central nervous system (such as the spinal cord, midbrain, hippocampus, or cortex), providing more accurate cell modeling for studying the mechanisms of BoNTs.

Embryonic stem cells (ESCs), with their multi-directional differentiation potential, can be directed to differentiate into neural lineage cells, such as functional motor neurons and astrocytes, and construct organoid models with neural synaptic networks and neurotransmitter release systems. These characteristics enable precise, in-vivo simulation of the neural microenvironment, thereby providing a detection platform with stronger physiological relevance for the toxicity detection of BoNTs [54]. When using ESCs to detect BoNTs, species differences also need to be considered. For example, the affinity of the human synaptotagmin II receptor for BoNT/B1 is different from that of the mouse synaptotagmin II receptor [25]. This difference can lead to higher results when using the embryonic stem cell model derived from mice to detect BoNT/B1 activity. The use of humanized embryonic stem cells can avoid the influence of inter-species differences on the test results. Research data indicate that human-induced pluripotent stem cells (hiPSCs) can be directed and differentiated into human motor neurons (MNs) expressing BoNT-specific receptors and substrates through three differentiation protocols. Comparative experiments showed that the detection sensitivity of MNs obtained by these three protocols for BoNT/A1 and BoNT/B1 was at least 30 times higher than that of MBA. However, protocol sensitivity variations may stem from receptor expression levels, protein concentrations, and electrophysiological properties during differentiation [55].

The standardization of cell culture and BoNT incubation conditions is a key factor in ensuring the repeatability of CBA. Cell sensitivity to BoNTs varies with incubation time, sample substrate, and toxin serotype. Some basic cell culture conditions are also important, such as culture temperature, pH value, and nutritional status. The above factors will affect the binding, internalization, translocation, and endopeptidase activity of toxins, thereby influencing the repeatability of the experiment. Some optimizations of culture conditions can effectively improve the detection performance of CBA. For example, pretreatment of cells with a high concentration of KCl can enhance the binding and internalization of toxins [27]. Adding glycolipids during culture can enhance the binding capacity of BoNT/B to synaptotagmin Ⅱ. The addition of ganglioside GT1b can promote the binding of BoNTs to receptors, thereby enhancing the sensitivity of neurons to toxins and improving the cleavage efficiency of BoNT/A [56].

The CBA method for BoNT bioactivity detection offers two principal advantages: rapid analysis and versatile endpoint analysis. Researchers can evaluate toxin activity either by quantifying intracellular SNARE protein cleavage products or by monitoring neurotransmitter release. To enhance physiological relevance, scientists have developed an NMJ model through co-culturing hiPSC-derived MNs with myotubes. The NMJ model replicates human neuromuscular interactions, providing a comprehensive platform for BoNT activity assessment with improved clinical predictability [45]. Real-time analysis of BoNT synaptic effects is achieved through electrophysiological techniques like patch clamp and calcium imaging, providing cell data with a high spatiotemporal resolution. In contrast to conventional methods focusing on primary toxin actions (e.g., cleavage of target proteins or blockade of neurotransmitter release), the NMJ model allows for systematic evaluation of the functional coupling between MN signaling and myotube contractile responses. This integrated approach directly reflects the ultimate biological effect of BoNTs: flaccid paralysis of muscle fibers resulting from neuromuscular transmission blockade.

Many studies have further verified the reliability of CBA in BoNT detection by combining it with other detection techniques. For example, by integrating CBA with bioluminescence technology, researchers enabled the expression of the NanoLuc-VAMP luciferase reporter system in SiMa cells and quantitatively detected BoNT/B by measuring the changes in luminescence signals caused by BoNT/B-mediated VAMP cutting [57]. Furthermore, its combination with neurotransmitter release monitoring technology can precisely quantify the inhibitory effect of toxins on synaptic transmission. More innovatively, researchers constructed the SNAP-25-Nanoluc recombinant reporter protein through molecular cloning technology. When BoNTs cut SNAP-25, the luciferase fragment dissociates, resulting in signal attenuation. This genetic engineering detection system based on the hiPSC-derived MN model can achieve femomolar-level detection sensitivity without antibodies [58].

At present, the FDA has approved the potency test of CBA for BoNT/A [48,53]. In our laboratory, we have completed the equivalence verification of the cell-based potency assay (CBPA) and the mLD50 for the efficacy determination of commercial BoNT/A (BOTOX). The objective was to explore the applicability of CBPA as a potential alternative for BoNT/A potency testing in China [59]. The results show that the CBPA meets all criteria and is equivalent to the mLD50 method, thereby improving detection efficiency while reducing the use of experimental animals.

## 4. Immunological Methods

Immunological analysis methods, based on the specific binding of antigens and antibodies, have become a primary technical means for BoNT detection. Among these methods, ELISA recognizes toxin epitopes through enzyme-labeled antibodies and achieves signal conversion via substrate color reactions, thereby enabling both qualitative and quantitative detection of BoNTs [60,61,62]. Due to its high detection sensitivity, standardized operational procedures, and cost-effectiveness, this method is widely used for high-throughput analysis in clinical diagnosis, food safety testing, and biomedical research. However, ELISA also has some technical limitations. Firstly, such methods fail to discriminate active or inactive toxins and are prone to interference from matrix effects, so inactivated toxins or interfering matrix components in samples may produce false positives. Secondly, Neurotoxin-Associated Proteins (NAPs), as key co-complexes of BoNTs, play an important role in maintaining the stability of toxins, penetrating the host barrier and promoting internalization [63,64]. However, their steric hindrance can mask the antigenic epitopes of BoNTs, thereby significantly inhibiting the antibody–antigen binding efficiency, which is also a common problem faced by other immunological detection methods. At present, some laboratories avoid such interferences by purifying and removing NAPs. Several newly developed and screened antibodies can recognize free epitopes on toxins, overcoming the problem of NAPs [64,65].

New high-sensitivity immunoassay technologies are constantly emerging, such as SpinDx, a centrifugal microfluidic immunoassay platform [66]. This technology specifically binds toxins through antibody-functionalized capture particles, uses the density medium in the microfluidic dish to settle these particle complexes, and combines a laser-induced fluorescence detection system to achieve quantitative analysis. The detection limit of SpinDx is as low as 0.09 pg/mL, achieving higher sensitivity than ELISA. It requires only 2 μL of an untreated sample and less than 30 min to complete the test. Furthermore, due to its unique centrifugal sedimentation and density medium separation mechanism, SpinDx effectively removes unbound labels and background interference from samples, enabling direct analysis of complex matrix samples such as whole blood, serum, and food.

Immuno-polymerase chain reaction (iPCR) is an innovative detection technology that combines nucleic acid reporter genes with specific detection antibodies. Antibody-bound BoNTs capture attached DNA fragments precisely, enabling efficient qPCR amplification for significant signal enhancement, achieving a sensitivity up to 10^5^ times higher than that of the traditional ELISA method. The iPCR method shows high efficacy in BoNT detection, with special advantages for trace-concentration antigen identification [67]. However, iPCR operation remains technically challenging due to the demanding operation and complicated synthetic coupling between the reporter gene and specific antibody.

**Figure 2:**
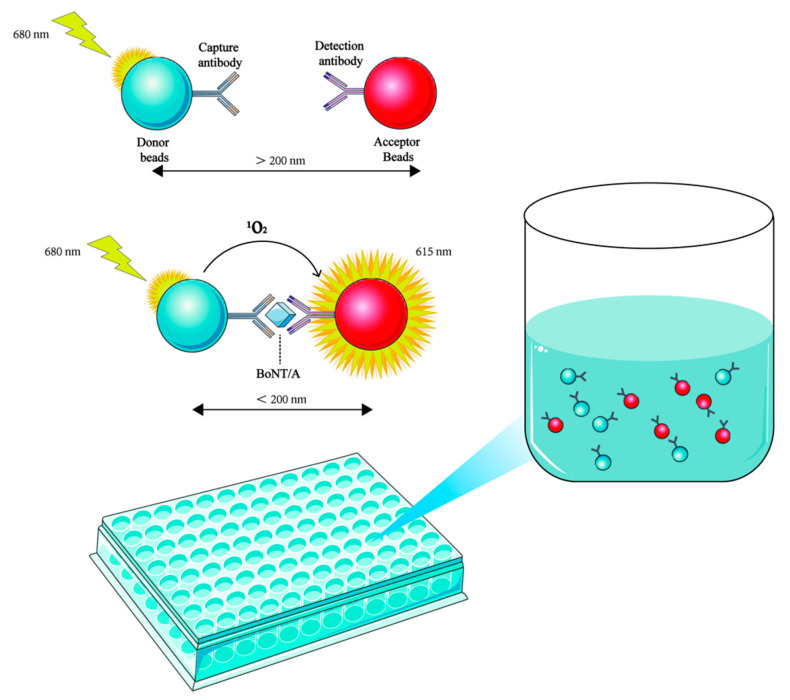
The schematic diagram of AlphaLISA demonstrates that donor beads generate singlet oxygen (^1^O_2_) upon excitation by a 680 nm laser. When BoNT is present, the donor beads form a tight complex (distance < 200 nm) with acceptor beads through capture antibodies and conjugated detection antibodies, enabling the diffusion of ^1^O_2_ from donor beads to acceptor beads and triggering a chemiluminescent reaction (emitting 615 nm fluorescence). When BoNT is absent, the donor beads and acceptor beads cannot generate optical signals due to their excessive distance.

The Amplified Luminescent Proximity Homogeneous Assay-Linked Immunosorbent Assay (AlphaLISA) is based on the specific binding of donor beads and acceptor beads. This homogeneous immunoassay technique has been successfully applied to the detection of BoNT/A in complex matrices [68]. The donor bead is coupled with streptavidin and can specifically bind to the biotinylated capture antibody, while the acceptor bead binds to the detection antibody. Activation of the chemiluminescent substance on the donor bead produces a singlet oxygen. The donor bead and the acceptor bead are pulled closer together when bound by the target antigen simultaneously, allowing the energy of the singlet oxygen to be transferred to the acceptor bead and triggering its light emission. The concentration of the target antigen in the sample is then determined by measuring the intensity of the luminescent signal (Figure 2). AlphaLISA detection can achieve a high sensitivity with a detection limit of 0.1 ng/mL within 30 min, greatly surpassing the traditional MBA in both speed and sensitivity. Unlike ELISA, this technology does not require solid-phase coating or repetitive washing steps. Instead, homogeneous detection is achieved through the reaction of microbeads in the solution, avoiding the incomplete washing problem caused by complex matrices in heterogeneous reactions. This homogeneous reaction design also reduces the possibility of cross-contamination caused by repeated lid opening, liquid transfer, and plate washing operations. This simplifies the operation process, reduces reagent consumption, and lowers the risk of human error, all of which enhance cost-effectiveness. In addition, it is also compatible with automated high-throughput analysis systems. This combination of accuracy, rapidity, and matrix tolerance makes AlphaLISA particularly valuable in high-throughput screening for food safety monitoring and clinical diagnosis. Currently, this method has been successfully applied to the detection of BoNT/A in vomitus samples from patients with food poisoning [68].

Flow cytometry utilizes fluorescation-encoded microspheres and an automated fluid system to achieve simultaneous detection of multiple toxins in complex matrices [69]. Compared to ELISA, flow cytometry offers superior microsphere-based toxin capture kinetics, enabling efficient low-concentration analyte enrichment. Additionally, it supports multiparameter analysis within a single sample, enabling the simultaneous analysis of different serotypes of BoNTs, significantly enhancing detection efficiency and high-throughput screening capabilities. However, the equipment cost of flow cytometry is relatively high, which to some extent limits its application in laboratories or on-site rapid detection with limited resources.

Lateral Flow Assays (LFAs) represent a well-established platform for rapid detection of BoNTs based on the principle of immunochromatography to enable point-of-care diagnostics. The traditional LFA uses colloidal gold-labeled antibodies to bind with BoNT molecules to form complexes, which are then migrated to the test line by chromatography and captured, thereby generating detection signals visible to the naked eye. This method has outstanding advantages such as low cost, simple operation, and rapid detection, but its sensitivity is limited, which to some extent restricts its wider practical application [70]. Some new materials have been used to overcome the drawback of low sensitivity. Notably, the development of surface-enhanced Raman scattering–lateral flow immunoassay (SERS-LFIA) biosensor strips based on SiO_2_@Au nanoparticles (NPs) has demonstrated a 100-fold enhancement in sensitivity than the colloidal gold LFA test strip; this advancement not only preserves the advantages of LFAs but also significantly elevates their analytical performance, opening new ways for highly sensitive on-site toxin detection [71].

## 5. Chromatography and Mass Spectrometry

Chromatography and mass spectrometry techniques have demonstrated unique technical advantages in the detection of BoNTs. Based on the combined strategy of immunoaffinity chromatography and liquid chromatography–tandem mass spectrometry (LC-MS/MS), researchers can precisely analyze the protein composition of the botulinum progenitor toxin complex (PTC) [72]. It is important to note that for the analysis of complex matrices, the exceptional sensitivity and specificity of MS often depend on a prior immunoaffinity enrichment step using antibodies to pull down and concentrate the toxin, thereby reducing background interference. Compared with traditional antibody-based methods, MS technology can achieve precise protein identification through peptide fingerprinting and can also decipher sequence features as well as critical post-translational modifications such as phosphorylation and glycosylation. This capability compensates for antibody-based detection limitations in discerning subtle protein structural differences.

This technology offers high sensitivity and specificity for BoNT subtype detection, with the detection cycle shortened to just a few hours [73]. A typical example is the successful application of LC-MS/MS for trace detection of BoNTs in honey samples, effectively preventing infant botulism [74]. In two cases of BoNT/A poisoning in France, the detection results from serum samples were highly consistent with MBA [75]. Size-exclusion chromatography (SEC) has also demonstrated unique value in the field of biopharmaceuticals. Its specific separation capabilities allow for precise determination of the high-molecular-weight forms and content of BoNT/A, providing a new solution for quality control [76]. In the study of NAPs, LC-MS/MS technology has systematically identified the presence of BoNT/G, non-toxic non-hemagglutinin (NTNH), hemagglutinin 70 (HA-70), HA-17, and the novel HA-33 protein in type G toxin complexes. With their precise analytical capabilities, chromatography and mass spectrometry have become essential tools for the detection, quantification, and mechanistic studies of BoNTs, driving the development of clinical diagnostics and food safety monitoring [77].

## 6. Endopeptidase–Mass Spectrometry

BoNTs exert their biological effects through endopeptidase activity, specifically cleaving substrates such as SNARE proteins. Based on this mechanism, the established endopeptidase–mass spectrometry (Endopep-MS) method can directly reflect the presence and activity of toxins by detecting the cleavage ability of BoNTs on specific peptide substrates [78,79,80]. Comparative studies have shown that the detection sensitivity of Endopep-MS is significantly higher than that of MBA, and it still has excellent detection ability at low toxin concentrations. Endopep-MS also demonstrates outstanding specificity, with no cross-reaction among serotypes. It can accurately identify the target serotype even in samples with high concentrations of non-target toxins or complex microbial backgrounds [81]. Compared to MBA, Endopep-MS avoids the ethical controversies of animal experiments and significantly reduces the testing time from a maximum of 4 days to within 8 h, greatly improving the testing efficiency. We believe that Endopep-MS is an accurate, efficient, and cost-effective BoNT detection method, which is highly suitable for the rapid response and confirmation of botulism poisoning outbreaks in public health laboratories and is expected to become the future standard method to replace MBA.

BoNTs are highly lethal proteins, and during botulism, the protein content of BoNTs in the samples to be tested is often very low. Moreover, the detection of BoNTs by Endopep-MS involves the use of mass spectrometry (MS), and overly complex sample matrices can interfere with the analytical performance. Therefore, it is necessary to enrich BoNTs from the sample matrix before analysis. This enrichment step not only increases the protein concentration of BoNTs but also reduces potential matrix effects, making the results more accurate and reliable [82]. Based on immunoaffinity chromatography techniques, researchers used monoclonal antibodies to perform immunoaffinity purification of BoNTs [83]. The purified samples were then added to peptide substrates, and the cleavage products of the peptide substrates were detected by MS. The results successfully identified biologically active BoNTs in the samples and distinguished between different serotypes.

Another study developed a multimer system based on single-domain antibodies (sdAbs) capable of specifically purifying various BoNT subtypes and chimeric forms. Single-domain antibodies, also known as variable heavy-chain domain (VHH) antibodies, contain only one variable heavy-chain domain (VHH) and represent the smallest known intact antigen-binding fragment, with a molecular weight merely one-tenth that of conventional antibodies. These antibodies not only exhibit superior affinity for BoNTs, enabling effective detection of trace toxins, but also benefit from their small size, which facilitates high-yield expression in Escherichia coli or yeast systems, allowing production of high-purity products through simplified purification processes. These characteristics make them ideal candidates for BoNT diagnostics and therapeutics. In a study, researchers successfully constructed 52 distinct VHH multimers using a yeast expression system. Experimental results demonstrated that these multimers could specifically recognize BoNT/C, D, and their chimeric variants (DC and CD); maintain stability under high-salt conditions; and be directly applied in Endopep-MS detection [84]. The VHH antibodies significantly expand the serotype coverage and detection throughput, making it an indispensable tool for multiplex BoNT detection. Its unique advantages perfectly address the limitations of traditional antibodies in complex detection. The combination of VHH antibodies and Endopep-MS detection system provides key technical support for the establishment of a multiplex BoNT detection system and is of great significance for the clinical diagnosis of botulism.

Another strategy for achieving multiple detection of BoNTs is to optimize the design of specific peptide substrates [85]. Since both BoNT/A and E target the SNAP-25 protein, but the cleavage sites are only 17 amino acids apart (Type A: Q197-R198; Type E: R180-I181), the peptide substrate has spatial competition within the BoNT/A catalytic domain, resulting in detection interference. Researchers successfully solved this steric hindrance problem by shortening the N-terminal of the BoNT/A peptide substrate, enabling the two substrates to simultaneously bind to different regions of the catalytic domain, thus achieving synchronous detection of BoNT/A and E [86].

The synthesis and optimization of peptide substrates is one of the key strategies to enhance the detection sensitivity of Endopep-MS. Through systematic substrate engineering modification, the performance of this method can be significantly enhanced in terms of cutting efficiency and anti-interference ability. Typical optimization strategies include (1) replacing the lysine at position 75 with ornithine to enhance substrate affinity and (2) implementing the S75A mutation to increase the yield of the C-terminal product. The Pep-19 substrate obtained through multiple rounds of optimization has increased the detection sensitivity of BoNT/G by approximately 60 times (compared to the original substrate) while maintaining excellent serotype specificity [87]. Secondly, in terms of anti-interference performance, by prolonging the N-terminal sequence and optimizing the internal amino acid composition, the substrate’s resistance to non-specific proteases has been significantly enhanced [88]. In combination with salt washing and the use of protease inhibitors, it can effectively suppress the background interference in complex samples such as the liver and significantly enhance the signal intensity of specific lysis products [89]. These optimization strategies have enhanced the detection sensitivity of Endopep-MS, reinforcing its position as a reliable and highly sensitive method for BoNT detection. The above strategies highlight that Endopep-MS, as a platform method that can be continuously optimized and improved, has significant potential for continuous evolution in the field of BoNT detection.

## 7. Biosensors

Biosensors are analytical tools that detect biomolecules/biochemical reactions by coupling biological elements with signal-transducing physical/chemical components. According to their working principles and the types of detected signals, biosensors can be roughly classified into optical biosensors, electrochemical biosensors, acoustic biosensors, and thermal sensors. In recent years, with the continuous development of biosensor technology, various types of biosensors have been developed for the rapid and sensitive detection of BoNTs, providing strong technical support for fields such as food safety, biosafety, and clinical diagnosis [33,34,90,91,92].

Optical biosensors are the most employed in BoNT detection. A study achieved quantitative detection of BoNT/A by monitoring the diffusivity changes of fluorescent microspheres. The method involves specific binding of BoNT/A to capture antibodies immobilized on fluorescent microspheres, followed by formation of immunocomplexes with detection antibodies labeled by Au NPs, which increases particle size and reduces diffusivity. The Brownian motion of particles was analyzed by using the microscopic imaging system and the spatial cross-correlation algorithm, and the quantitative detection of BoNT/A was achieved by measuring the change in diffusibility. This strategy is quite novel and can to a certain extent avoid the light scattering interference of background fluorescence or complex matrices, thereby enhancing the reliability of detection in real samples. This approach demonstrated excellent performance across various matrices including milk, serum, and PBS, achieving a detection limit of 10 pg/mL with a rapid assay time of merely 2 min. Notably, the method maintains consistent performance regardless of medium viscosity, making it suitable for practical detection scenarios [35]. In addition to detecting the protein content of toxins, this method can be further developed or optimized by introducing SNARE substrates to assess the enzymatic activity of BoNTs, which can better reflect the true toxicity of BoNTs than simple protein quantity detection.

Another optical sensor employs two adjacent porous silicon Fabry–Pérot interferometers (FPIs) to detect BoNT/C via a dual mechanism of competitive immunoassay and specific substrate hydrolysis, with a detection limit of 4.24 pg/mL. In the competitive immunoassay, the BoNT/C toxoid fixed on the porous silicon surface competes with the toxins in the sample to bind specific antibodies. Subsequently, horseradish peroxidase (HRP)-labeled secondary antibodies are added to catalyze the generation of insoluble products, which fills the pores and alters the refractive index, resulting in changes in effective optical thickness (EOT) inversely proportional to toxin concentration. For specific substrate hydrolysis, immobilized SNAP-25/VAMP-2 substrates are cleaved by BoNT/C, releasing peptide fragments and reducing the pore refractive index. Here, EOT variations correlate positively with toxin enzymatic activity [93]. In this detection technology, competitive immunoassay and specific substrate hydrolysis are not two independent detection methods. While quantifying BoNT/C with high sensitivity, it can also test the protease activity of BoNT/C. This synergistic effect and complementarity make the results more convincing than those of single-toxin protein detection, and cross-validation with opposite signal change directions in the two detection modes can further reduce false positive or false negative results. The design of this sensor platform is not only applicable to BoNT/C. After replacing the antibodies and substrates, it has full potential to be extended to the detection of BoNTs in other serotypes, which can greatly enhance the breadth and influence of the work.

A study has developed a localized surface plasmon resonance (LSPR) nanosensor for the rapid detection of BoNTs based on Langmuir–Blodgett (LB) film preparation technology. The researchers employed LB to orderly deposit AuNPs on a polyethylene terephthalate (PET) substrate and then modified it with BoNT-specific antibodies. When the toxin binds to the antibody, the change in the surface chemical environment of the LB film triggers an LSPR signal (wavelength shift and absorbance change), thereby enabling quantitative detection of BoNTs. By adjusting the surface pressure during the preparation of the LB film, the arrangement of AuNPs and the thickness of the film can be precisely controlled, thereby optimizing the sensing capability of the sensor [94]. The study results indicate that the detection limit of the LB film prepared at a surface pressure of 35 mN/m is as low as 1 pg/mL. However, excessive surface pressure leads to the formation of AuNP clusters, resulting in reduced sensitivity.

A fluorescent biosensor based on Förster resonance energy transfer (FRET) between quantum dots (QDs) and dark quenchers-labeled peptide probes has been developed for the detection of BoNTs. The probe contains a specific cleavage site for BoNT/E, and in the presence of the toxin, it is specifically cleaved, resulting in changes in the photoluminescence (PL) intensity of the QDs. The sensor does not rely on antibodies, which reduces costs and enhances stability, and also avoids the possible cross-reaction problems caused by antibodies. It outperforms MBA in terms of sensitivity and detection time. The detection limits for BoNT/E light chain (LcE) and BoNT/E are 0.02 ng/mL and 2 ng/mL, respectively, with a detection time of only 3 h. By optimizing probe design and QD selection, the sensor could be extended to simultaneous detection of multiple BoNT serotypes, providing an efficient and sensitive solution for food safety and biodefense [95].

A paper-based electrochemical biosensor shows good applicability for detecting BoNTs in real matrices. The method is based on the detection principle of square wave voltammetry (SWV). A peptide similar to SNAP-25 was synthesized and labeled with the electroactive molecule methylene blue (MB), which was then immobilized on a paper-based electrode modified with AuNPs. When BoNT/A and BoNT/C cleave the peptide, MB is removed from the electrode surface, resulting in a decrease in the current signal, which is proportional to the concentration of BoNTs. The sensor output exhibits a good linear relationship for BoNT/A and BoNT/C in the concentration range of 0.01 to 1 nM, with a detection limit of 10 pM. In the application test with orange juice samples, the sensor demonstrated good recovery rates, indicating that the platform is capable of measuring BoNTs in real matrices [96]. However, this promising technology still shows certain limitations that need to be addressed in practical applications. Firstly, although the detection limit of 10 pM is considerable, it is still higher than the trace levels required for the detection of certain clinical or environmental samples and may not meet the detection requirements of extremely low concentration samples. Secondly, this method requires a relatively long incubation time (4 h) to obtain measurable signal changes, which is still rather lengthy for on-site rapid screening scenarios. At present, this platform only demonstrates the detection capability for serotypes A and C, while other clinically relevant serotypes have not yet been addressed. Moreover, when the paper-based electrode was stored under moist conditions, the detection signal showed a significant decrease on the 14th day, suggesting that the stability of the material needs to be improved.

The acoustic sensor based on surface acoustic wave (SAW) is capable of rapidly detecting the light chain protein of BoNT/A (LcA). By immobilizing specific antibodies on a gold electrode, the sensor monitors the mass changes induced by the binding of LcA through phase shifts in the acoustic wave. The sensor can complete detection within minutes and exhibits good repeatability. However, a significant limitation of the current system is that it uses only the LcA as the detection target, rather than the full-length BoNT/A. Since the molecular weight of the light chain is only about 50 kDa, this simplification may overestimate the sensitivity, as the larger mass and steric hindrance of the complete toxin would reduce the binding efficiency, which directly affected its overall detection capability. Under the current circumstances, this sensor shows low sensitivity, with a minimum detection limit of 0.5 μg/mL. The nonlinear correlation between phase shift and LcA concentration is also challenging for detection, and its overall performance is still lower than that of ELISA [97]. Despite these limitations, the successful application of this technology has fully demonstrated the feasibility of using SAW for rapid detection of LcA in liquid samples. Future improvements, such as optimizing the immunoassay format or introducing porous surface layers, could enhance its performance and provide a potential method for on-site screening.

A study introduces an innovative method that integrates wearable temperature sensors with gas sensors to detect botulism. The approach is based on the principle that botulism alters respiratory metabolism and body temperature. By screening five volatile organic compounds (VOCs) in exhaled breath as biomarkers, the system enables real-time monitoring of these markers alongside temperature fluctuations. The results demonstrate that this method achieves an accuracy exceeding 91.2% in early detection of botulism symptoms, providing warnings within 1.5 h post-exposure. This non-invasive, highly sensitive technique offers a novel tool for early botulism detection, facilitating timely clinical intervention [98].

## 8. Discussion

Current detection methods exhibit distinct advantages but retain limitations in information acquisition. For example, while immunological methods can effectively identify the presence of BoNTs, they cannot determine whether the toxin is biologically active [52]. Moreover, different immunological methods are suitable for different detection scenarios. LFAs, despite their low sensitivity, are still widely used for rapid preliminary screening due to their ease of operation and cost-effectiveness [99]. Flow cytometry is more suitable for applications that require rapid, high-throughput analysis and the simultaneous detection of multiple biomarkers. The most commonly used ELISA is cost-effective and easy to operate, with a detection sensitivity sufficient for most scenarios [31]. Therefore, selection of the detection method requires comprehensive consideration of detection needs, costs, equipment availability, and anticipated analysis complexity. In the future, the development of immunological methods should also take into account how to reduce the risk of false positives that may occur during the detection process. Based on existing experience, false positives mainly result from three types of mechanisms: First, matrix interference may be caused by non-specific proteases, lipids, or other components in complex sample matrices. For instance, in some immunoassay methods, matrix components may non-specifically bind to capture antibodies or detection antibodies, generating false positive signals. Secondly, the specificity of the antibody itself is insufficient. If the antibody cross-reacts with other non-target proteins in the sample, it can trigger false positive signals. Some bacteria can express proteins structurally similar to BoNT, or degradation products in the sample can expose conformations similar to toxin epitopes, all of which intensify this risk. Thirdly, the toxin proteins that have been inactivated in the samples after heating treatment may still be detected as “positive”, leading to an incorrect assessment of the actual toxicity.

In recent years, biosensors have become one of the most promising technologies for BoNT detection, as their core advantage lies in the ability to directly convert biological events such as antibody-antigen-specific binding or enzymatic digestion reactions into quantifiable physical signals, thereby achieving extremely high detection sensitivity and rapid response capability [100,101]. The advantages of biosensors also benefit from the development and application of functionalized nanomaterials and molecular recognition elements. For instance, gold nanoparticles are used to enhance signal amplification to improve sensitivity, and surface plasmon resonance (SPR) technology is employed to monitor the interaction between toxins and receptors in real time and without labels, significantly reducing the detection time. More significantly, these devices can be integrated into portable detection terminals via chip integration technology, enabling on-site rapid detection. However, the development of biosensors from innovative technologies to standardized detection methods still faces severe challenges. Non-specific components in the sample matrix adsorb onto the sensor surface, leading to signal drift, reduced sensitivity, and false positive results. Therefore, efficient and reliable sample pretreatment techniques have become an indispensable prerequisite in practical applications. In addition, most high-performance biosensors are still in the “principle verification” stage at present. The probes, nanomaterials, and sample processing methods adopted in different studies vary significantly, making it difficult to replicate and compare the results horizontally. There is also a lack of large-scale, multi-center clinical sample verification. In conclusion, the future development of biosensor technology must pursue high sensitivity while focusing on solving the above-mentioned key bottlenecks to promote its wider practical application.

CBA is the most promising alternative to MBA for detection to the best of our knowledge. Its primary advantage lies in the fact that it is not merely an immuno-binding assay but rather a functional detection platform capable of fully simulating the entire molecular pathological process of BoNTs in the body, from binding and internalization to substrate protein cleavage, thereby more directly reflecting the actual toxicity of the BoNTs. CBPA has been certified by the regulatory agency for quality control of BoNT/A, further validating its effectiveness [38,53]. CBA can also serve as an ideal platform for studying the toxicological mechanisms of BoNTs [102]. More importantly, CBA enables inhibitor and neutralizing antibody screening, which is critical for the development of novel therapeutic strategies and drugs [103]. However, CBA still has certain limitations at present. The entire detection process, including cell culture, toxin incubation, and signal detection, usually still takes several days and has a low degree of automation, making it difficult to achieve the high-throughput screening capacity of methods like ELISA or biosensors. Furthermore, an ideal CBA requires the use of neuronal cell lines that express specific receptors for BoNT, and the culture conditions, passage number, and differentiation state of these cells can significantly affect receptor expression levels and cellular sensitivity, leading to inter-experimental variation. Therefore, establishing a stable, uniform, and reproducible cell model remains a significant challenge. Future development of CBA should focus on developing more stable and standardized engineered cell lines and promoting the automation and miniaturization of detection steps, aiming to increase detection throughput and the robustness of the overall system.

With the study of the genetic sequences of BoNTs, the discovery of chimeric toxins, such as BoNT/FA and BoNT/CD, has driven the evolution of the traditional serotype classification framework. Meanwhile, more subtypes of each toxin serotype have been identified [16,104,105]. For example, BoNT/A and BoNT/B can be further divided into BoNT/A1 to A8 and BoNT/B1 to B8, which further highlights the evolutionary complexity of BoNTs [106]. The continuous emergence of these chimeric toxins and new subtypes indicates that the diversity of BoNTs far exceeds current understanding. This diversity poses a dual challenge to future detection technologies: while developing a broad-spectrum detection system is crucial for recognizing emerging chimeric toxins, equally important is the creation of high-resolution techniques to accurately discriminate between subtypes. Consequently, next-generation detection platforms must combine broad serotype identification with high-resolution subtype discrimination to effectively monitor continuously evolving toxin variants.

## Data Availability

No new data were created or analyzed in this study.
